# Value of single-center fecal calprotectin in the early diagnosis and assessment of necrotizing enterocolitis in premature infants

**DOI:** 10.3389/fped.2025.1598448

**Published:** 2025-07-03

**Authors:** Chen ZongLi, Luo KeYong, Jiang Liang

**Affiliations:** Department of Neonatology, The First People’s Hospital of Zunyi, Zunyi, China

**Keywords:** premature infants, necrotizing enterocolitis, fecal calprotectin, early diagnosis, disease assessment, assessment

## Abstract

**Objective:**

To explore the value of fecal calprotectin (FC) in the early diagnosis of necrotizing enterocolitis (NEC) in premature infants.

**Methods:**

From September 2021 to June 2024, 84 premature infants with NEC were selected as the NEC group, and 84 premature infants with feeding intolerance (feeding intolerance group) and 168 healthy premature infants (healthy group) were selected at the same time. ROC curves were used to analyze the value of FC in the early diagnosis and condition evaluation of NEC in premature infants, and Spearman correlations were used to analyze the relationships between FC and the occurrence and severity of NEC in premature infants.

**Results:**

FC levels in the NEC group were greater than those in the feeding intolerance group and healthy group (*P* < 0.05), and there was no significant difference between the feeding intolerance group and the healthy group (*P* > 0.05). The FC level of premature infants with NEC III was greater than that of premature infants with NEC Ⅰ and Ⅱ, and the FC level of premature infants with NEC II was greater than that of premature infants with NEC Ⅰ (*P* < 0.05). ROC curve analysis revealed that the best diagnostic values of FC for premature infants with NEC and their conditions were 8.40 μg/g and 53.50 μg/g, respectively, and the AUCs were 0.651 and 0.901, respectively, with sensitivities of 42.86% and 85.71%, specificities of 89.23% and 82.61%, respectively. Spearman correlation analysis revealed that FC was positively correlated with the occurrence and severity of NEC in premature infants (*r*_s_ = 0.401, 0.853; *P* < 0.05).

**Conclusion:**

The level of FC in premature infants with NEC is abnormally high, and FC has high clinical value for early diagnosis and condition evaluation of premature infants with NEC, which is worthy of further investigation.

## 1 Introduction

Necrotizing enterocolitis (NEC) is an extremely serious gastrointestinal emergency in the neonatal period that seriously threatens the lives of preterm infants. Even if infants survive, they are likely to have sequelae such as short bowel syndrome, intestinal stenosis, and abnormal neurological development (1), early diagnosis and timely treatment of NEC are highly important for reducing the mortality rate of NEC and improving its prognosis. The main clinical manifestations include abdominal distension, vomiting, diarrhea, and bloody stools. In severe cases, shock and multiple organ dysfunction occur. The characteristic finding on abdominal x-ray examination is pneumatosis intestinalis (2). Epidemiological investigations have shown that the incidence of NEC in the neonatal intensive care unit (NICU) is 2%–5%, among which the mortality rate is 23%–30% (3). With the development of neonatal intensive care technology, the survival rate of preterm infants has significantly increased, but the incidence of NEC is on the rise (4). Owing to the diverse clinical manifestations of NEC, which are difficult to distinguish from other diseases, such as feeding intolerance and neonatal sepsis (5). In addition, biological indicators such as C-reactive protein and procalcitonin lack specificity and sensitivity for the early identification of NEC, which is likely to lead to missed diagnosis and misdiagnosis, delaying treatment (6). Early diagnosis remains challenging. Therefore, exploring new biomarkers is the focus of the current early diagnosis and disease assessment methods for NEC. Fecal calprotectin (FC) is a 36.5-kDa calcium and iron-binding protein that accounts for 60% of the soluble proteins in human neutrophils and is present mainly in monocytes, macrophages, and epithelial cells (7). FC can reflect the activation state of white blood cells, and its expression level in feces is greater than that in peripheral blood (8). Studies (9) have shown that patients with inflammatory bowel disease and gastrointestinal bacterial infections have a relatively high concentration of FC in their feces, and the concentration of fecal FC in patients with acute bacterial infections is the highest. At present, many reports at home and abroad have discussed the value of FC in the diagnosis of NEC, but there is still controversy about its diagnostic value. In this study, by comparing and analyzing FC levels in preterm infants with NEC and those with feeding intolerance, the value of FC in the early diagnosis of NEC and its severity in preterm infants was explored to provide a reference basis for clinical application.

## 2 Materials and methods

### 2.1 General information

A total of 89 preterm infants with NEC were admitted to the hospital from September 2021 to June 2024. According to the inclusion and exclusion criteria, 84 preterm infants with NEC were ultimately included. At the same time, 84 preterm infants with feeding intolerance and 168 preterm infants without complications were selected. The NEC group included 37 males and 47 females; the average gestational age was 32.00 ± 2.61 weeks; the average birth weight was 1,475.70 ± 69.61 g; and the delivery methods were as follows: 60 cases by cesarean section and 24 cases by spontaneous delivery. In the feeding intolerance group, there were 49 males and 35 females; the average gestational age was 31.20 ± 2.46 weeks; the average birth weight was 1,521.20 ± 51.40 g; and the delivery methods were as follows: 65 cases by cesarean section and 19 cases by spontaneous delivery. In the healthy group, there were 96 males and 72 females; the average gestational age was 32.66 ± 2.54 weeks; the average birth weight was 1,538.11 ± 43.57 g; and the delivery methods were as follows: 131 cases by cesarean section and 37 cases by spontaneous delivery. There were no significant differences in general information among the three groups (*P* > 0.05), indicating comparability. According to the modified Bell staging criteria in practical neonatology (10), the NEC group was divided into 3 stages, with 25 patients in stage I, 44 patients in stage II, and 15 patients in stage III. There were no significant differences in general information among the preterm infants with NEC at the three stages (*P* > 0.05), indicating comparability see Table 1.

**Table 1:** Comparison of the general information of preterm infants with NEC at different stages.

Group	*n*	Gender (*n*, Male/Female)	Gestational age ($\bar{x}\pm s$, weeks)	Birth weight ($\bar{x}\pm s$, g)	Delivery method (*n*, Cesarean section/spontaneous delivery)
Stage Ⅰ	25	14/11	31.09 ± 2.19	1,457.00 ± 68.21	18/7
Stage Ⅱ	44	19/25	32.67 ± 2.81	1,472.08 ± 65.48	32/12
Stage Ⅲ	15	4/11	32.29 ± 2.43	1,511.29 ± 65.30	10/5
*Z*/*F*		0.531	1.196	1.457	0.292
*P*		0.767	0.318	0.251	0.864

### 2.2 Inclusion criteria

(1) Gestational age at birth <37 weeks. (2) Feeding intolerance: Preterm infants present one or more of the following symptoms after enteral feeding: gastric retention (gastric residual volume >2 ml/kg), abdominal distension, and vomiting more than 3 times a day. In severe cases, symptoms of gastrointestinal bleeding, such as coffee-ground-like gastric contents, positive fecal occult blood, melena or bloody stools, occur. (3) NEC: Suspected symptoms such as vomiting, abdominal distension, diarrhea, and mucus bloody stools can occur. Combined with the results of abdominal x-ray, puncture or surgery, the diagnostic and staging criteria refer to the modified Bell staging criteria in the 5th edition of practical neonatology (10). (4) Healthy group: Preterm infants without genetic metabolic diseases, with gastrointestinal symptoms, and with smooth feeding.

### 2.3 Exclusion criteria

(1) Congenital gastrointestinal developmental malformations, such as congenital hypertrophic pylorus, intestinal malrotation, and intestinal atresia; (2) chromosomal abnormalities and genetic metabolic diseases. (3) Congenital developmental diseases such as congenital heart disease. (4) The specimen submission time exceeds 24 hours.

### 2.4 Methods

#### 2.4.1 Specimen collection

When a child is clinically suspected of having NEC or feeding intolerance, feces collection begins. At the same time, feces from one preterm infant without complications was collected. Each fecal sample was approximately 0.5–1.0 g, and the FC concentration was detected.

#### 2.4.2 Specimen storage

The fecal specimen was placed in a routine EP tube for submission, sealed, and then stored frozen in a refrigerator (−20°C).

#### 2.4.3 Specimen detection

The detection should be carried out as soon as possible after collection and should not exceed 24 hours. The fecal samples were removed from the refrigerator and thawed at room temperature. The samples were subsequently centrifuged at 5,000 r/min for 5 min, the supernatant was collected, and the FC concentration was detected via colloidal gold immunochromatography. The fecal calprotectin kits were all purchased from Xiamen Weizheng Biotechnology Co., Ltd. All the samples were detected by the same device.

### 2.5 Observation indicators

(1) The FC levels of preterm infants in the NEC group, the feeding intolerance group, and the healthy group were compared. (2) The FC levels of preterm infants with NEC at different stages were compared. (3) The ROC curve of the FC concentration in the NEC group was drawn, and the optimal diagnostic threshold of FC for NEC in preterm infants was calculated. (4) To analyze the correlation between FC and the occurrence and severity of NEC in preterm infants.

### 2.6 Statistical analysis

The data were statistically analyzed via SPSS 27.0 software. Normally distributed data are expressed as the mean ± standard deviation ($\bar{x}\pm s$). One-way analysis of variance was used for comparisons among the three groups; the FC levels of the three groups are expressed as the interquartile range [M (P25, P75)], and nonparametric tests were used for comparisons among groups. Enumeration data are expressed as n, and the chi-square test or rank sum test was used. The ROC curve was used to analyze the diagnostic value of fecal FC for NEC and its severity in preterm infants. Spearman correlation analysis was used to analyze the correlation between fecal FC and NEC and its severity in preterm infants; *P* < 0.05 was considered to indicate statistical significance.

## 3 Results

### 3.1 Comparison of FC levels among the three groups

The FC level in the NEC group was greater than that in the feeding intolerance group and the healthy group (*P* < 0.05). There was no significant difference in the FC level between the feeding intolerance group and the healthy control group (*P* > 0.05) see Table 2.

**Table 2:** Comparison of FC among the three groups [M (P25, P75), μg/g].

Group	*n*	FC
NEC group	84	31.90 (65.73, 197.19)
Feeding intolerance group	84	9.90 (8.05, 24.15)
Healthy group	168	8.75 (15.83, 47.49)
*H*		20.400
*P*		<0.001

**Figure 1:**
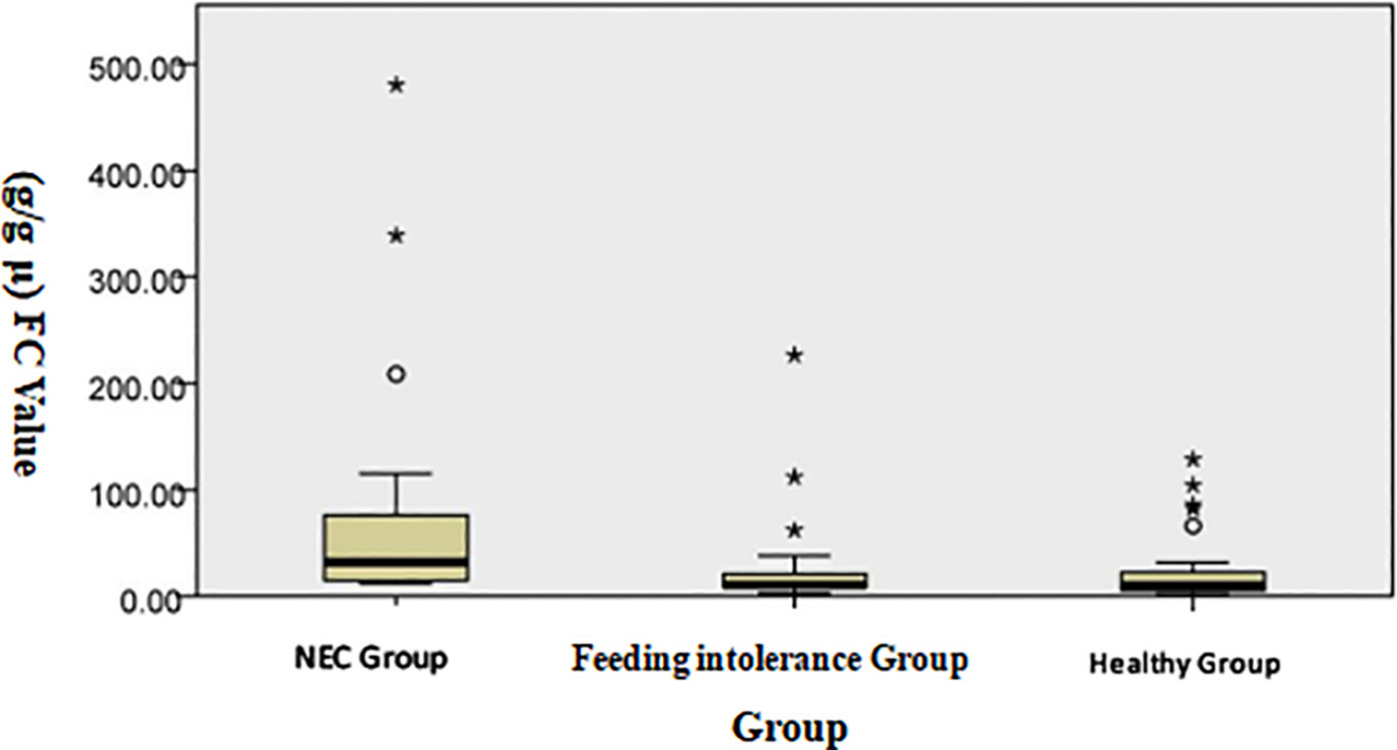
Distribution of FC levels among the groups (the Kruskal–Wallis test was used for independent samples).

**Figure 2:**
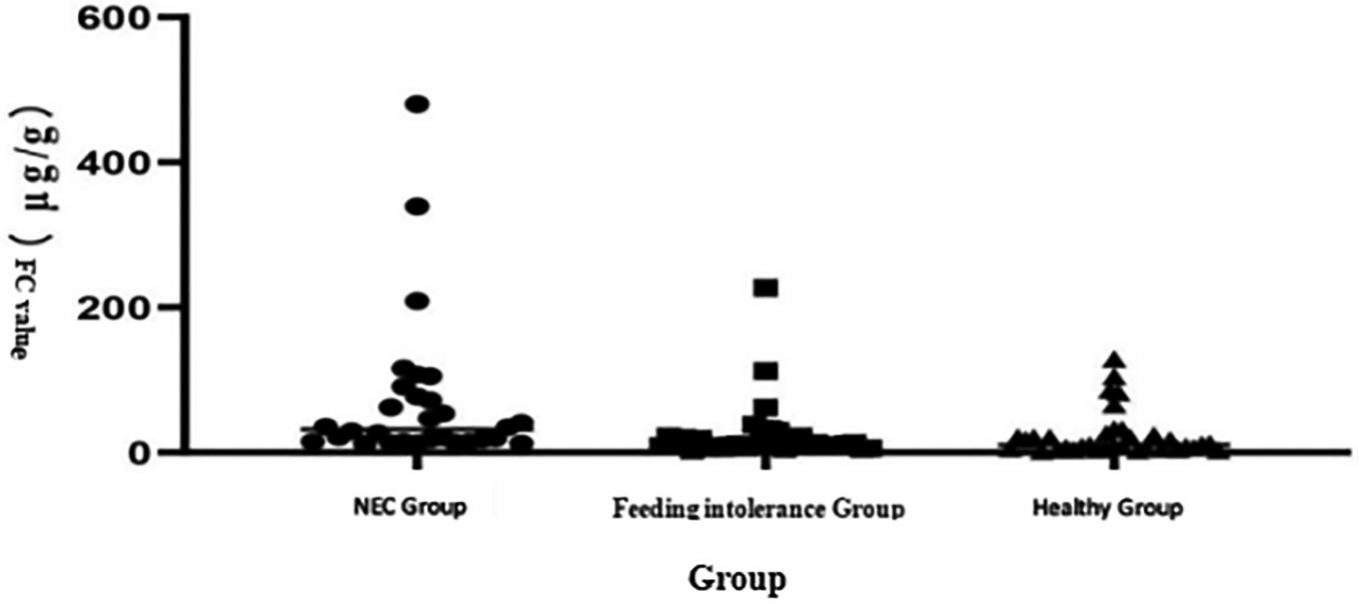
Scatter plot of FC levels among the groups.

The analysis of the three groups through the Kruskal–Wallis test shows that the median level of FC in the NEC group was significantly higher than that in the feeding intolerance and healthy groups, as shown in Figure 1. Scatter plot distribution: Although there was overlap among the three groups, the FC level in the NEC group was generally higher than that in the feeding intolerance and healthy groups, as shown in Figure 2.

### 3.2 Comparison of FC levels in NEC children at different stages

The FC level of children with stage III NEC was greater than that of children with stage I and stage II NEC, and the FC level of children with stage II NEC was greater than that of children with stage I NEC (*P* < 0.05) see Table 3.

**Table 3:** Comparison of FC levels in NEC children at different stages (μg/g).

NEC Stage	*n*	FC
Stage Ⅰ	25	14.83 ± 2.61
Stage Ⅱ	44	54.42 ± 31.72
Stage Ⅲ	15	187.54 ± 167.01
*F*		10.066
*P*		0.001

### 3.3 ROC curve analysis of FC in the diagnosis of NEC in preterm infants

**Figure 3:**
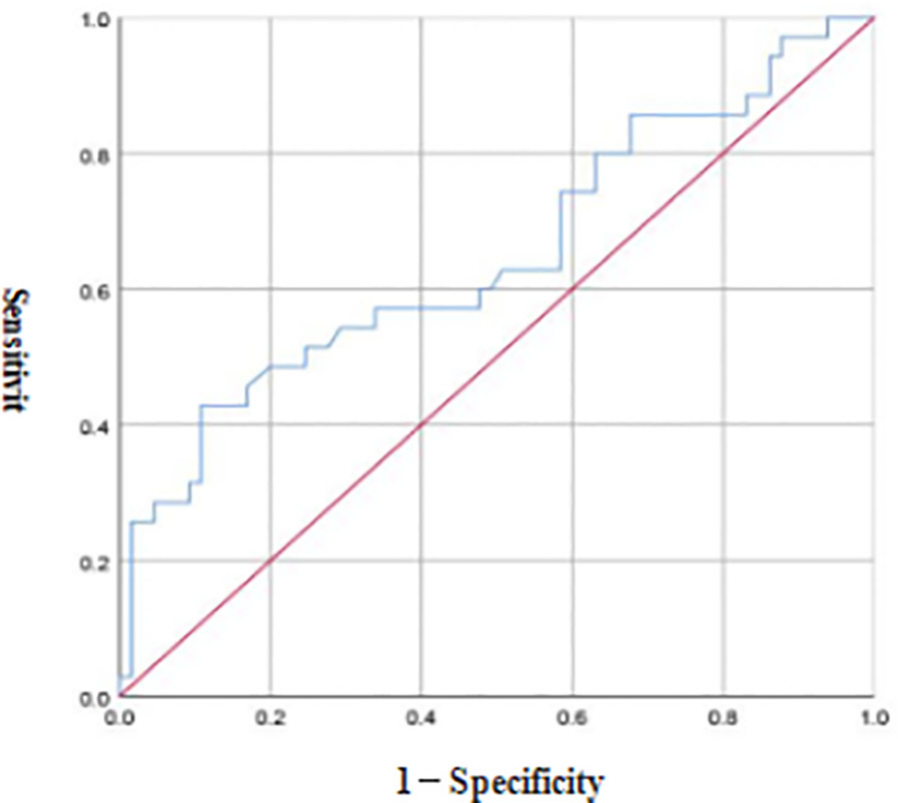
ROC curve of FC in the diagnosis of NEC in preterm infants.

ROC curve analysis revealed that the optimal diagnostic threshold of FC for NEC in preterm infants was 8.40 μg/g, the AUC was 0.651, and the diagnostic sensitivity and accuracy were 42.86% and 89.23%, respectively see Figure 3.

### 3.4 ROC curve analysis of FC in the differential diagnosis of NEC in preterm infants at different grades

**Figure 4:**
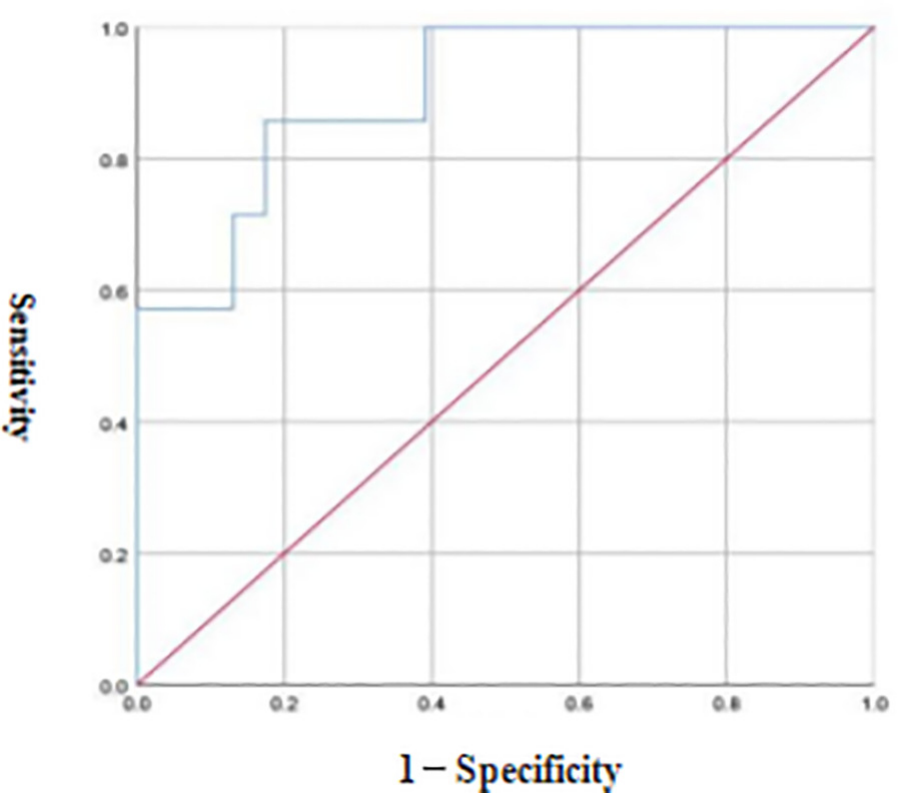
ROC curve of FC in the differential diagnosis of NEC in preterm infants at different grades.

ROC curve analysis revealed that the optimal diagnostic threshold of FC for the severity of NEC in preterm infants was 53.50 μg/g, the AUC was 0.901, and the diagnostic sensitivity and accuracy were 85.71% and 82.61%, respectively see Figure 4.

### 3.5 Spearman correlation analysis between FC and NEC in preterm infants

Spearman correlation analysis revealed that FC was positively correlated with the occurrence and severity of NEC in preterm infants (*P* < 0.05) see Table 4.

**Table 4:** Spearman correlation analysis between FC and NEC in preterm infants.

Correlation	FC	
	*r_s_*	*P*
NEC	0.401	<0.001
NEC stage	0.858	<0.001

## 4 Discussion

NEC is a unique type of intestinal necrotizing inflammation in the neonatal period that mostly occurs in preterm infants and has a high mortality rate. Currently, its exact pathogenesis has not been fully elucidated. Previous studies (11) have shown that the occurrence of NEC may be related to a reduction in the intestinal mucosal blood supply, ischemia and hypoxia, improper feeding, or bacterial infection in children and that its progression can lead to intestinal rupture, peritonitis, sepsis, and even death. At present, the clinical diagnosis of NEC depends on a series of clinical symptoms, laboratory indicators, and imaging findings. The above clinical parameters are not specific and cannot provide sufficient evidence for the early diagnosis of NEC. In addition, there is currently no specific treatment plan for NEC, and its prevention strategies are only intended to encourage the use of breast milk, the application of probiotics, early prevention, feeding management, etc. (12). Therefore, exploring early specific biomarkers of NEC is helpful for early diagnosis and timely intervention, thereby improving the prognosis.

FCs are effective neutrophil chemotactic factors. When there is inflammation in the intestinal tract of the body, neutrophils exude into the intestinal cavity, leading to an increase in the FC level (13, 14). Preterm infants have increased intestinal permeability due to factors such as the tight junctions of intestinal epithelial cells, which is conducive to the exudation of neutrophils into the intestinal cavity. In addition, FC detection is noninvasive and simple, which greatly reduces stimulation in preterm infants. These characteristics make it a reliable biomarker for screening for NEC. A number of studies (15–17) have shown that FC has high clinical application value in the diagnosis of inflammatory bowel disease in children and NEC in neonates. The results of this study revealed that the FC level in the NEC group was greater than that in the feeding intolerance group and the healthy group, and there was no significant difference in the FC level between the feeding intolerance group and the healthy group, suggesting that the FC level in preterm infants with NEC was significantly greater and that FC is expected to become an early diagnostic indicator for NEC in preterm infants. The reason for this finding is that NEC may irreversibly damage the entire intestinal tract of the child, and a large-scale intestinal mucosal infection leads to an increase in the FC level. When FC binds to zinc ions, a stable structure, which has good heat resistance, is formed. This characteristic makes FC difficult to degrade in feces. Therefore, the FC level in preterm infants with NEC is significantly greater than that in preterm infants with feeding intolerance and normal preterm infants. Studies (18) have shown that FC has high specificity and sensitivity in the diagnosis of NEC in preterm neonates. The results of the ROC curve analysis in this study revealed that the optimal diagnostic value of FC for NEC in preterm infants was 8.40 μg/g, the AUC was 0.651, and the diagnostic sensitivity and accuracy were 42.86% and 89.23%, respectively, indicating that FC has a certain diagnostic value for NEC in preterm infants. The results of the Spearman correlation analysis in this study also revealed that FC was positively correlated with the occurrence of NEC in preterm infants, further indicating that the FC level is closely related to the occurrence of NEC in preterm infants. This may be related to the induction of neutrophil exudation into the intestinal cavity by intestinal inflammation. Therefore, monitoring FC levels has certain clinical value for the early diagnosis of NEC in preterm infants.

Studies (19) have shown that FC has high diagnostic value for the severity of NEC in neonates. The results of this study revealed that the FC level of preterm infants with NEC at stage III was greater than that of preterm infants at stages I and II, and the FC level of preterm infants with NEC at stage II was greater than that of preterm infants at stage I, indicating that the higher the clinical stage of preterm infants with NEC is, the greater the FC level. The possible reason for this finding is that the immune system of preterm infants is immature and that the tight junctions of intestinal epithelial cells increase intestinal permeability, leading to the exudation of neutrophils into the intestinal cavity. Moreover, the degree of intestinal inflammation in preterm infants with stage III NEC is greater than that in preterm infants at stages I and II, and the degree of neutrophil exudation into the intestinal cavity is greater, which leads to a high FC. Reports (20) have noted that dynamic monitoring of the FC level can reflect the occurrence and development of necrotizing enteritis in preterm infants to a certain extent. The results of the ROC curve analysis in this study revealed that the optimal diagnostic value of FC for the severity of NEC in preterm infants was 53.50 μg/g, the AUC was 0.901, and the diagnostic sensitivity and accuracy were 85.71% and 82.61%, respectively, indicating that FC has high diagnostic value for the severity of NEC in preterm infants. The results of the Spearman correlation analysis in this study also revealed that FC was positively correlated with the severity of NEC in preterm infants, indicating that the FC level is related to the development of NEC in preterm infants.

Limitations of this study: (1) Owing to the sharp decline in the birth rate, improvement in perinatal health care technology levels, increase in the breast milk feeding rate of preterm infants in the NICU, and failure to collect and submit some samples of children in a timely manner, the sample size included in this study was small. (2) Some studies have shown that the FC concentration is related to the day of age, which may affect the test results.

## 5 Conclusion

The FC level in preterm infants with NEC is abnormally high. Moreover, FC is noninvasive and has intestinal specificity, which can largely compensate for the disadvantages of current blood test methods, such as high degree of trauma and poor specificity. It has good clinical application value for the early diagnosis and disease assessment of NEC in preterm infants and is worthy of further research. Since all the patients included in this study were preterm infants with NEC admitted to our hospital, the sample size was limited, and the FC level in preterm infants can also be affected by other factors (21, 22). In the future, multicenter, large-sample experiments should be carried out to further confirm its diagnostic efficiency and provide a theoretical basis for its promotion and application.

## Data Availability

The original contributions presented in the study are included in the article/Supplementary Material, further inquiries can be directed to the corresponding author.

## References

[1] Maheshwari A Traub TM Garg PM Ethawi Y Buonocore G. Necrotizing enterocolitis: clinical features, histopathological characteristics, and genetic associations. Curr Pediatr Rev. (2022) 18(3):210–25. 10.2174/1573396318666220204113858 35125082

[2] Evidence-based Professional Committee of Neonatologists Branch of Chinese Medical Doctor Association. Clinical practice guidelines for neonatal necrotizing enterocolitis (2020). Chine J Contemp Pediatr. (2021) 23(1):1–11. 10.7499/j.issn.1008-8830.2011145

[3] Cao X Zhang L Jiang S Li M Yan C Shen C Epidemiology of necrotizing enterocolitis in preterm infants in China: a multicenter cohort study from 2015 to 2018. J Pediatr Surg. (2022) 57(3):382–6. 10.1016/j.jpedsurg.2021.05.014 34175121

[4] Lin H Mao S Shi L Tou J Du L. Clinical characteristic comparison of low birth weight and very low birth weight preterm infants with neonatal necrotizing enterocolitis: a single tertiary center experience from eastern China. Pediatr Surg Int. (2018) 34(11):1201–7. 10.1007/s00383-018-4339-9 30128701

[5] Kim JH Sampath V Canvasser J. Challenges in diagnosing necrotizing enterocolitis. Pediatr Res. (2020) 88(Suppl 1):16–20. 10.1038/s41390-020-1090-4 32855507

[6] Dong H Zhang L Li B Li J Chen Y Richard SA Screening inflammatory protein biomarkers on premature infants with necrotizing enterocolitis. Inflamm Res. (2023) 72(4):757–68. 10.1007/s00011-023-01702-6 36806964 PMC10129932

[7] Jukic A Bakiri L Wagner EF Tilg H Adolph TE. Calprotectin: from biomarker to biological function. Gut. (2021) 70(10):1978–88. 10.1136/gutjnl-2021-324855 34145045 PMC8458070

[8] Shimizu H Ebana R Kudo T Sato T Hara T Hosoi K Both fecal calprotectin and fecal immunochemical tests are useful in children with inflammatory bowel disease. J Gastroenterol. (2022) 57(5):344–56. 10.1007/s00535-022-01856-w 35165800

[9] Krishnakumar C Ananthakrishnan AN Boyle BM Griffiths AM LeLeiko NS Mack DR Early change in fecal calprotectin predicts one-year outcome in children newly diagnosed with ulcerative colitis. J Pediatr Gastroenterol Nutr. (2022) 74(1):72–8. 10.1097/MPG.0000000000003291 34433783

[10] Xiaomei S Hongmao Y Xiaoshan Q. Practical Neonatology. 5th ed. Beijing: People’s Medical Publishing House (2019). p. 632–9.

[11] Gagné D Shajari E Thibault MP Noël JF Boisvert FM Babakissa C Proteomics profiling of stool samples from preterm neonates with SWATH/DIA mass spectrometry for predicting necrotizing enterocolitis. Int J Mol Sci. (2022) 23(19):11601. 10.3390/ijms231911601 36232903 PMC9569884

[12] Chandran S Anand AJ Rajadurai VS Seyed ES Khoo PC Chua MC. Evidence-based practices reduce necrotizing enterocolitis and improve nutrition outcomes in very low-birth-weight infants. JPEN J Parenter Enteral Nutr. (2021) 45(7):1408–16. 10.1002/jpen.2058 33296087

[13] Stevens TW Gecse K Turner JR de Hertogh G Rubin DT D'Haens GR. Diagnostic accuracy of fecal calprotectin concentration in evaluating therapeutic outcomes of patients with ulcerative colitis. Clin Gastroenterol Hepatol. (2021) 19(11):2333–42. 10.1016/j.cgh.2020.08.019 32801008 PMC8140548

[14] Gacesa R Vich Vila A Collij V Mujagic Z Kurilshikov A Voskuil MD A combination of fecal calprotectin and human beta-defensin 2 facilitates diagnosis and monitoring of inflammatory bowel disease. Gut Microbes. (2021) 13(1):1943288. 10.1080/19490976.2021.1943288 34313538 PMC8317932

[15] Jinglin X Bingbing L Ruiquan W Lianqiang W Dongmei C Bin W. Changes and clinical significance of fecal calprotectin levels in very low birth weight infants. Chin Pediatr Emerg Med. (2021) 28(10):890–4. 10.3760/cma.j.issn.1673-4912.2021.10.010

[16] Ying Z Xu J. Clinical significance of fecal calprotectin in children with inflammatory bowel disease. Lab Med. (2022) 37(5):429–32. 10.3969/j.issn.1673-8640.2022.05.006

[17] Yan-Qiu X Chang-Jun R Xiong W Shu-Wen X Xiao-Xing W Ling H. Meta-analysis of the diagnostic role of fecal calprotectin in neonatal necrotizing enterocolitis. Chin J Contemp Pediatr. (2021) 23(4):381–9. 10.7499/j.issn.1008-8830.2010111 PMC8050553 33840411

[18] Thibault MP Tremblay É Horth C Fournier-Morin A Grynspan D Babakissa C Lipocalin-2 and calprotectin as stool biomarkers for predicting necrotizing enterocolitis in premature neonates. Pediatr Res. (2022) 91(1):129–36. 10.1038/s41390-021-01680-7 34465872 PMC8770124

[19] Lingying H Cunfang B. Value of fecal calprotectin combined with intestinal barrier function in the grading diagnosis of neonatal necrotizing enterocolitis. Mater Child Health Care China. (2023) 38(7):1242–5. 10.19829/j.zgfybj.issn.1001-4411.2023.07.022

[20] van Zoonen AGJF Hulzebos CV Muller Kobold AC Kooi EMW Bos AF Hulscher JBF. Serial fecal calprotectin in the prediction of necrotizing enterocolitis in preterm neonates. J Pediatr Surg. (2019) 54(3):455–9. 10.1016/j.jpedsurg.2018.04.034 29859621

[21] Ran T Jun Y Xiaohua W Yuejing X Bomei Y. Correlation between changes in fecal calprotectin levels after birth in neonates and perinatal and postnatal clinical factors. Mater Child Health Care China. (2023) 38(13):2367–70. 10.19829/j.zgfybj.issn.1001-4411.2023.13.010

[22] Yiwei Z Yafei G Shudong C Ye Z Xiaosong S. Dynamic changes of fecal calprotectin within 2 weeks after birth in preterm infants and its influencing factors. Chin J Evid Based Pediatr. (2015) 10(05):357–60. 10.3969/j.issn.1673-5501.2015.05.007

